# Soft Inductive Coil Spring Strain Sensor Integrated with SMA Spring Bundle Actuator

**DOI:** 10.3390/s21072304

**Published:** 2021-03-25

**Authors:** Kyungjun Choi, Seong Jun Park, Mooncheol Won, Cheol Hoon Park

**Affiliations:** 1 Department of Robotics and Mechatronics, Korea Institute of Machinery & Materials, Daejeon 34103, Korea; choikj@kimm.re.kr (K.C.); sjpark61@kimm.re.kr (S.J.P.); 2 Department of Mechatronics Engineering, Chungnam National University, Daejeon 34103, Korea; mcwon@cnu.ac.kr; 3 Department of Mechanical Engineering, Sungkyunkwan University, Suwon 16419, Korea

**Keywords:** soft strain sensor, inductive sensor, coil spring sensor, SMA spring bundle actuator, soft actuator

## Abstract

This study proposes a soft inductive coil spring (SICS) strain sensor that can measure the strain of soft actuators. The SICS sensor, produced by transforming a shape memory alloy (SMA) wire with the same materials as that of an SMA spring bundle actuator (SSBA) into a coil spring shape, measures inductance changes according to length changes. This study also proposes a manufacturing method, output characteristics of the SICS sensor applicable to the SSBA among soft actuators, and the structure of the SICS sensor-integrated SSBA (SI-SSBA). In the SI-SSBA, the SMA spring bundle and SICS sensor have structures corresponding to the muscle fiber and spindle of the skeletal muscle, respectively. It is demonstrated that when a robotic arm with one degree of freedom is operated by attaching two SI-SSBAs in an antagonistic structure, the displacement of the SSBA can be measured using the proposed strain sensor. The output characteristics of the SICS sensor for the driving speed of the robotic arm were evaluated, and it was experimentally proven that the strain of the SSBA can be stably measured in water under a temperature change of 54 °C from 36 to 90 °C.

## 1. Introduction

Human–robot collaboration is being widely applied in the industrial and service fields, and active research is being conducted to develop robots that can be operated safely in close proximity to humans. In particular, studies are being conducted to secure intrinsic safety by developing safe, light, and compliant robotic arms by applying lightweight artificial muscles instead of the existing motor and gear mechanism. Among the artificial muscles applicable to robotic arms, the shape memory alloy (SMA) spring bundle actuator (SSBA) was recently developed [1]. The SSBA can contract and relax with the changing water temperature using a faucet-like valve, and force control is possible by adjusting the water temperature. For example, the SSBA of a 12 g mass consisting of 24 SMA coil springs and a highly stretchable silicone tube can actuate with a contraction strain of more than 50% and actuating frequency of 1 Hz under a mass condition of 10 kg, and can generate 130 N of force based on the water temperature change from 28 to 82 °C. The SSBA may be used for developing a lightweight safe robotic arm because it has a contraction rate higher than that of skeletal muscles, as well as high load capacity, fast actuation frequency, and force and position controllability.

**Figure 1:**
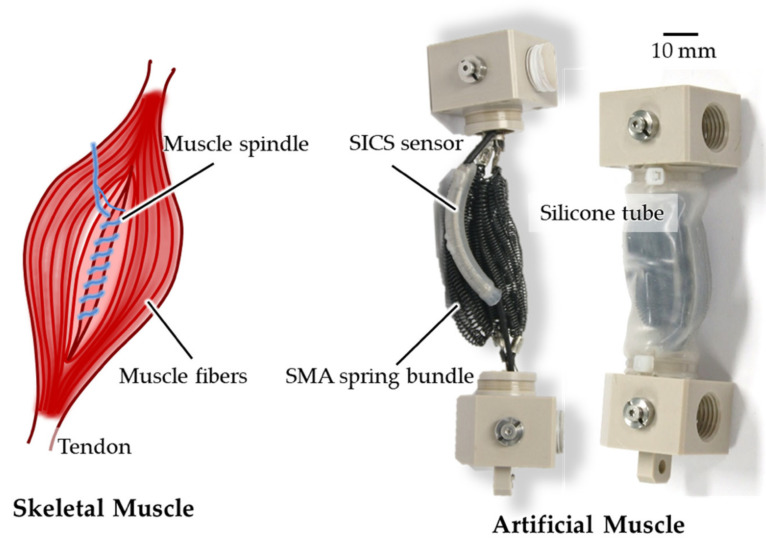
Shape memory alloy (SMA) spring bundle actuator integrated with a soft inductive coil spring (SICS) strain sensor that mimics skeletal muscles, including muscle fibers and muscle spindle.

As shown in Figure 1, skeletal muscles are bundles of muscle fibers, and SSBA consists of a bundle of SMA springs that mimic the skeletal muscles. A strain sensor is required to control the displacement of an SSBA by measuring and feeding back the contraction and relaxation lengths of the SSBA. The strain sensor of the SSBA must satisfy a few requirements. The SSBA can be relaxed by up to 200% compared with the contracted state. Therefore, the strain sensor must be soft, flexible, and highly stretchable like the SSBA, and its structure must be integrated with the SSBA and be capable of contracting or relaxing according to the changing length of the SSBA. The SSBA is activated by temperature changes in water between 20 and 90 °C generated by mixing hot and cold water. Therefore, the strain sensor must have stable output characteristics, even with a wide range of temperature changes in the SSBA.

Existing sensors for linear displacement, such as linear variable differential transformers (LVDTs) and wire encoders, are unsuitable for small flexible SSBAs because they are rigid and large. The research on soft sensors that can measure large displacements is as active as the research on soft actuators. Until now, soft strain sensors of resistive and capacitive types have been mainly researched, and resistive soft strain sensors using liquid metals and silicone have been extensively researched. These sensors, which measure the strain by sensing changes in electrical resistance, show high sensitivity and stretchability [2,3,4,5,6,7,8,9,10,11,12,13,14]. However, they are complex to produce, have large nonlinearity and hysteresis, and are highly sensitive to temperature changes. As a result, these sensors cannot be easily used through a simple calibration only [4,8,9,10,15,16,17,18]. Because SSBAs are activated by water in the temperature range of 20–90 °C, it is unsuitable for application to resistive strain sensors that are sensitive to temperature changes [15]. A silicone-based capacitive soft strain sensor has better linearity and hysteresis characteristics than a resistive soft strain sensor, but it is not easy to produce thin and stretchable electrodes [19,20]. Furthermore, because silicone is expanded or shrunk by temperature variation, which changes the sensitivity and output signal drift, it is unsuitable for SSBAs, which show rapid and large temperature changes [19,21,22,23]. The amount of flexion of the optical fiber changes according to the change in the length or flexion of the soft actuator, and the optical soft strain sensor based on the principle of measuring changes in the intensity of light transmitted through the fiber has also been researched [24,25,26,27,28]. Optical sensors have the advantages of good linearity and producibility, but they are unsuitable for SSBAs with large temperature changes because of the low sensitivity and large variations in characteristics according to temperature changes [26,28].

This paper proposes a soft inductive coil spring (SICS) strain sensor based on the principle that the inductance changes according to changes in the length of the SMA spring of the SSBA. An SMA spring that is identical to the one used in the SSBA can be integrated into the SSBA by combining it with the SMA spring bundle of the SSBA as an SICS sensor. This study also suggests a manufacturing method for the SICS sensor, presents the results of linearity evaluation, and proposes a structure of an SICS sensor-integrated SSBA (SI-SSBA). Our findings demonstrated that when a robotic arm with one degree of freedom (DOF) is operated by attaching two SI-SSBAs in an antagonistic structure by supplying hot and cold water, the displacement of the SSBA can be measured using the proposed strain sensor. This finding confirms that the proposed SMA spring strain sensor combined with the SSBA can stably measure large displacements in water with large temperature changes.

## 2. Operation Principle of the SICS Sensor

**Figure 2:**
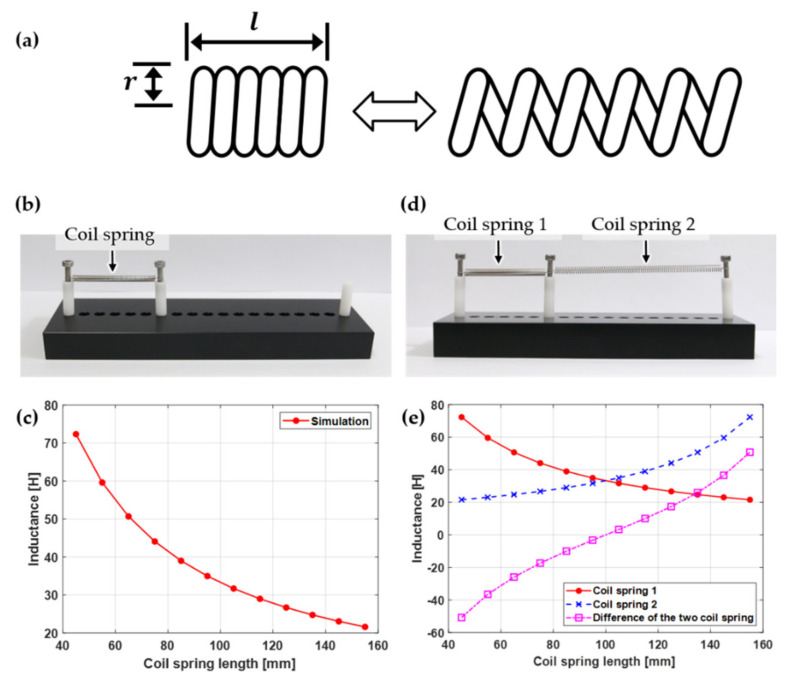
(**a**) Geometry of coil spring. (**b**) Single coil spring and (**c**) its inductance depending on the length of coil spring. (**d**) Two coil springs and (**e**) their differential inductance change depending on the length of coil springs.

The inductance of the coil spring with a sufficient length, as shown in Figure 2a, can be expressed as Equation (1) [29], where $\mu_{0}$ is the magnetic permeability of the free space ($4\pi \times 10^{-7}{\mathrm{Hm}}^{-1}$); $\mu_{r}$ is the relative permeability of the core (1 for air core); and N, l, and r are the number of turns, length, and radius of the coil spring, respectively. If the coil inside is filled with air, then the inductance is determined by the geometry of the coil [30] from Equation (1).

(1) $$L\left[H\right]=\mu_{0}\mu_{r}\left(\frac{N^{2}}{l}\pi r^{2}\right)$$

Figure 2c shows the result of predicting the inductance change using Equation (1) when a coil spring composed of stainless steel is lengthened in 10 mm intervals, as shown in Figure 2b. The longer the length of the coil spring, the lower the inductance, but the inductance change for the same length change tended to decrease.

For a coil spring used as a strain sensor with uniform sensitivity according to displacement, its linearity can be improved by measuring the inductance difference of two coil springs after connecting two coil springs in series so that their length changes would occur in opposite ways. For example, when coil springs 1 and 2 are placed as shown in Figure 2d, as the length of coil spring 1 is increased at fixed intervals, the length of coil spring 2 is also reduced in the same intervals. If the difference in the inductance between coil springs 1 and 2 is calculated, then a sensor output with improved linearity can be obtained, as shown by the pink line in Figure 2e.

The two SSBAs applied to the robotic arm were placed in an antagonistic structure, similar to the biceps and triceps of the upper arm. Thus, when one muscle is contracted, the other muscles are relaxed. Therefore, two SICS sensors embedded in two SSBAs operate in different types according to the flexing–extending motions of the robot arm. Consequently, the motions of the robotic arm can be measured with high linearity, as shown in Figure 2e, by measuring the difference in inductance between the two SICS sensors.

**Figure 3:**
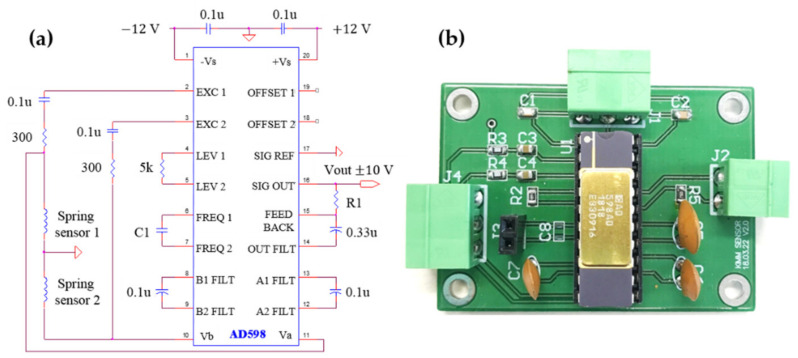
Half-bridge circuit of AD598: (**a**) schematic diagram and (**b**) fabricated board.

To measure the output of the differential-type SICS sensors, a sensor amplifier was fabricated using the AD598, an LVDT signal conditioner from Analog Devices Inc. [31]. Originally, the AD598 outputs the relative positional changes between an LVDT core and two coils with a high-accuracy voltage. However, in this study, the AD598 and two coil springs were configured in a half-bridge circuit, which was used to measure the inductance changes resulting from the difference in the lengths of two coil springs. The voltage output of the AD598 was measured by connecting it to a data acquisition (DAQ) board (PCI-6221, National Instruments). The half-bridge circuit of the AD598 is shown in Figure 3. The gain and offset of the output voltage can be adjusted by controlling the excitation capacitor C1 and resistance R1 of the output terminal.

## 3. Design and Fabrication of the SICS Sensor

The coil spring used as the SICS sensor was fabricated with an SMA wire of the same material and spring diameter as those of the SMA spring applied to the SSBA. The SMA coil spring of the SSBA was fabricated in 52 turns, and its length was increased to 133 mm under a maximum load of 10 kg. However, the inductance of the coil spring for the sensor decreases in the form of an exponential function as the length increases in a limited number of turns. Therefore, the range of the inductance change can be improved by selecting the maximum possible number of turns. In this study, the SMA coil spring for the SICS sensor was fabricated in 116 turns of 58 mm corresponding to the maximum length for embedding in the SSBA to maximize the linearity of the coil spring. Table 1 summarizes the specifications of the SMA coil spring for the SICS sensor.

**Figure 4:**
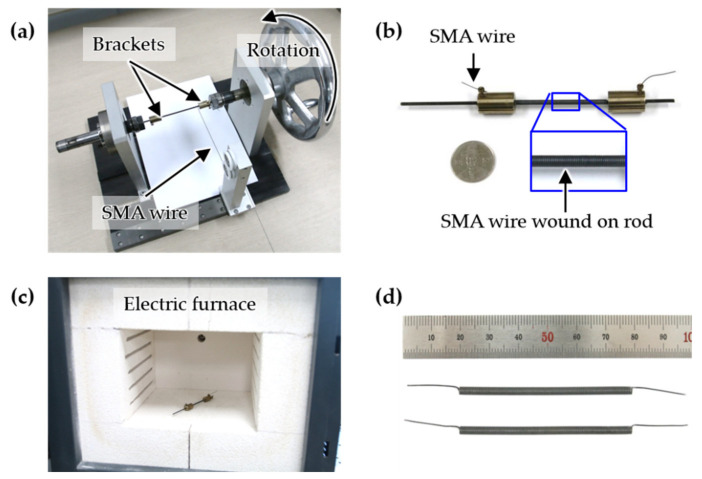
Fabrication process of the SMA coil for the SICS sensor: (**a**) winding jig, (**b**) SMA wire wound on the rod, (**c**) electric furnace for annealing, and (**d**) fabricated SMA coil for the SICS sensor.

The fabrication process of the coil spring for the SICS sensor is shown in Figure 4. A rod with an outer diameter of 2 mm and two brackets were fixed to the winding jig, as shown in Figure 4a. After fixing the SMA wire with a diameter of 0.5 mm to one side of the bracket, the SMA wire was wound for the required number of turns by rotating the handle. The other end of the SMA wire was fixed to the bracket of the other side to prevent loosening of the wound SMA wire (Figure 4b). When the fixed SMA wire wound on the rod was annealed for 30 min at 350 °C in an electric furnace, an SMA coil spring memorized in a coil form was fabricated, as shown in Figure 4d [1].

## 4. Output Characteristics of the SICS Sensor

**Figure 5:**
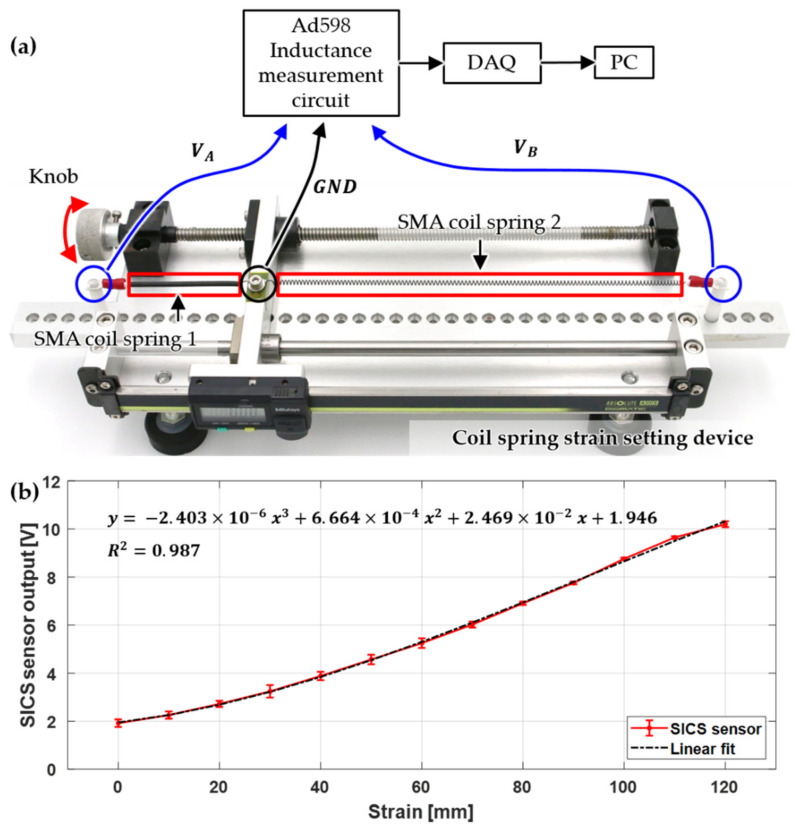
(**a**) Experimental configuration for measuring the relationship of the strain and output of SICS sensors and coil strain setting device and (**b**) measured relationship between the strain and sensor output.

The voltage output of the AD598 sensor amplifier for the strain of the SMA coil springs was measured using the experimental setup shown in Figure 5a. When the knob of the coil spring strain setting device was rotated, the ground (GND) part to which the ends of two springs were connected was moved, and the extension and contraction of the same strain occurred in the two SMA coil springs. The AD598 circuit detects the strain of the spring in the coil spring strain-setting device and outputs an analog voltage. A value of 10 pF was used for C1 of the AD598 sensor amplifier and 45 kΩ for R1. The voltage measured in the DAQ was sent to a PC.

As shown in Figure 5a, the position where coil spring 1 was contracted to the maximum and coil spring 2 was extended to the maximum was set as the reference point of the 0 mm strain. In addition, the output voltage was measured while setting the strain to generate extension and contraction in 10 mm increments between coil springs 1 and 2. This experiment was repeated five times under the same conditions. Figure 5b shows the output voltage for the strain of the coil springs. From the small range-of-error bar, it can be seen that the outputs were repeatable. For a strain of 120 mm, a voltage of approximately 8 V was applied. The relationship between the output voltage and strain was expressed as a third-order polynomial using the least squares method, as shown in Equation (2), which showed a high correlation with a coefficient of determination ($R^{2}$) of approximately 0.987. Thus, the output voltage can be converted to a strain of the coil spring using the following calibration equation:

(2) $$L_{diff}\left(x\right)=a_{3}x^{3}+a_{2}x^{2}+a_{1}x+a_{0}$$

where $a_{3},a_{2},a_{1},$ and $a_{0}$ are the coefficients of the third-order least squares polynomial.

## 5. Evaluation of the SICS Sensor in a Robotic Arm

**Figure 6:**
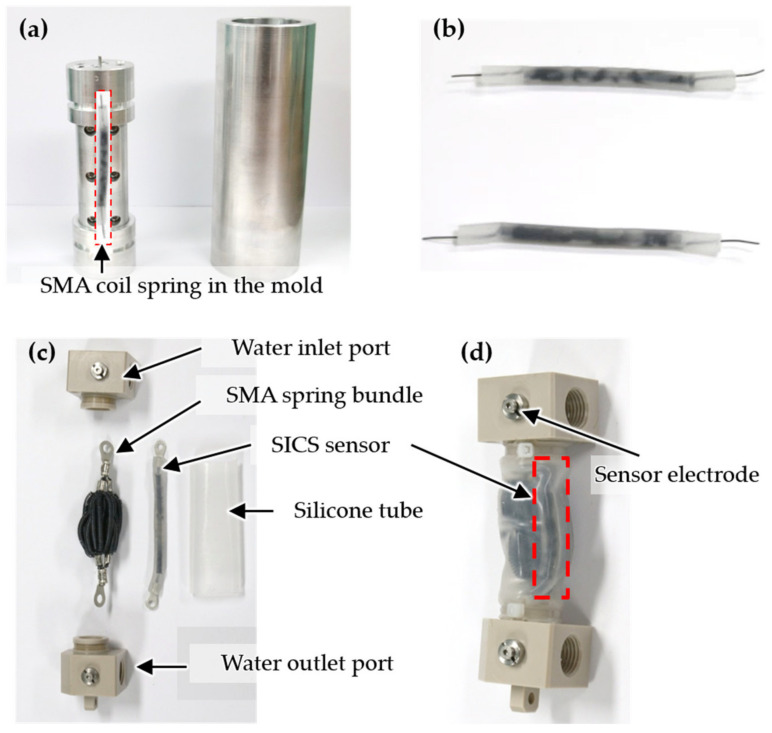
(**a**) Molds for silicone molding on the SMA coil; (**b**) silicone-molded SMA coils; (**c**) components before integrating the SMA spring bundle actuator (SSBA) and SICS sensor; (**d**) SICS sensor-integrated SSBA (SI-SSBA).

Because the SSBA is composed of a bundle of SMA coil springs, if the SMA coil springs for the SICS sensor are directly combined with the SSBA, then the measurement of inductance becomes unstable owing to the contact of the SSBA with the SMA coil springs. To avoid this, the SMA coil spring for the SICS sensor must be insulated. The SMA coil springs for the SICS sensor need to be repetitively extended and contracted according to the stroke of the SSBA. Hence, the coil spring was insulated by molding the SMA coil spring with silicone (Ecoflex 00-30, Smooth-On, Inc., Macungie, PA USA), which is the same material as the tube of the SSBA. The insulated SMA coil spring was fabricated, as shown in Figure 6b, by molding it to form a silicone tube outside of the SMA coil spring using the frame shown in Figure 6a. Because the silicone insulator for the SMA coil spring is highly stretchable, as is the tube of the SSBA, it stretches and contracts according to the displacement of SSBA.

Then, the SI-SSBA was fabricated. The SSBA is composed of an SMA spring bundle, a silicone tube, and water inlet and outlet ports, as shown in Figure 6c. Twenty-four SMA springs were used in the SSBA, and the detailed specifications of the SMA springs are presented in Reference [1]. After inserting the SMA spring bundle and SICS sensor together inside the silicone tube, they were assembled with water inlet and outlet ports. An electrode for connecting the SICS sensor to the outside was added, as shown in Figure 6d. In the SI-SSBA, the SMA spring bundle and SICS sensor have a structure that corresponds to the muscle fiber and muscle spindle of the skeletal muscle, as shown in Figure 1.

**Figure 7:**
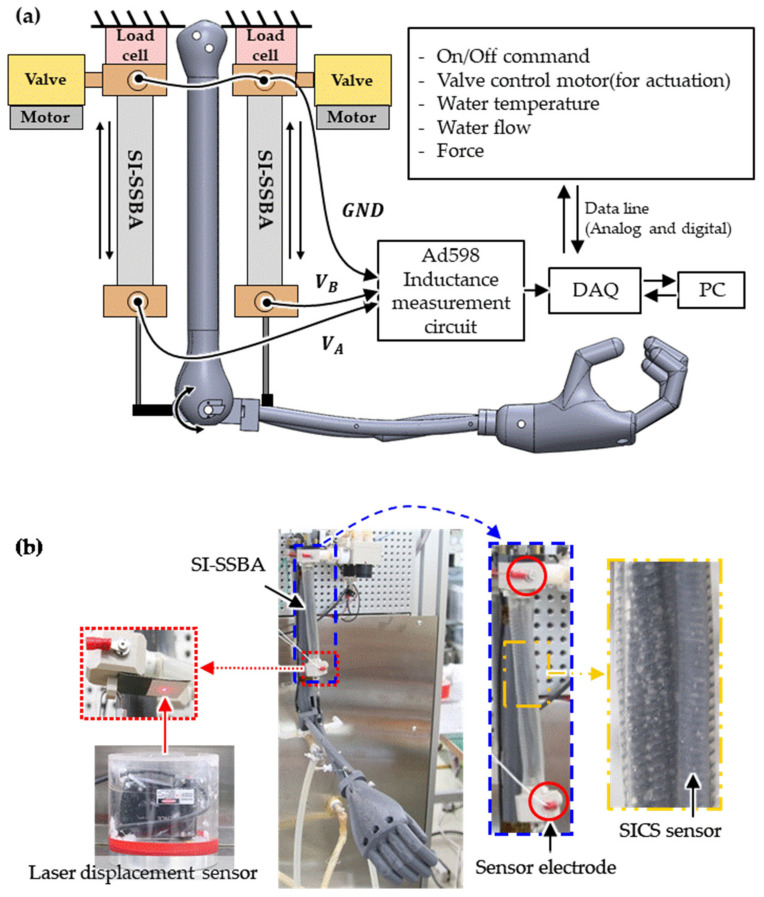
(**a**) Experimental configuration and (**b**) setup for actuating the robotic arm and measuring its angular displacement.

To evaluate the measurement performance of the SICS sensor for the motions of the SI-SSBA in which the actuator and strain sensor were integrated, an experimental setup was configured, as shown in Figure 7. The SSBA was replaced with the SI-SSBA in the experimental setup presented in Reference [1]. The 1 DOF robotic arm that can move the elbow joint is composed of an upper arm, forearm, and hand by simulating the skeletal structure of the human arm and was fabricated using a 3D printer. The SI-SSBAs were attached to an antagonistic structure at positions corresponding to the biceps and triceps of the upper arm. The hot and cold water were mixed to the required temperature at the valve, and the mixed water was supplied to the SI-SSBA. The SMA spring bundle inside the SI-SSBA was contracted or extended according to the temperature of the supplied water, and the robotic arm was flexed and extended accordingly (Video S1).

To verify the performance of the SICS sensor, the output voltage was measured using the SICS sensor for the strain of the SI-SSBAs while contracting and relaxing by supplying water to the SI-SSBAs. At the same time, the strain of the SI-SSBA was measured as a reference by measuring the displacement of the aluminum target attached to the bottom of the water outlet port of the biceps SI-SSBA using the laser displacement sensor at the bottom. It was then compared with the output of the SICS sensor. The robotic arm was extended to the maximum when 36 °C water was supplied to the biceps SI-SSBA and 90 °C water was supplied to the triceps SI-SSBA. The strain of the SI-SSBA measured by the laser displacement sensor at this time was set to 0 mm. In addition, the robotic arm was flexed by contracting the SI-SSBA while gradually adjusting the water temperature. By contrast, the robotic arm was flexed to the maximum when 90 °C water was supplied to the biceps SI-SSBA and 36 °C water was supplied to the triceps SI-SSBA.

**Figure 8:**
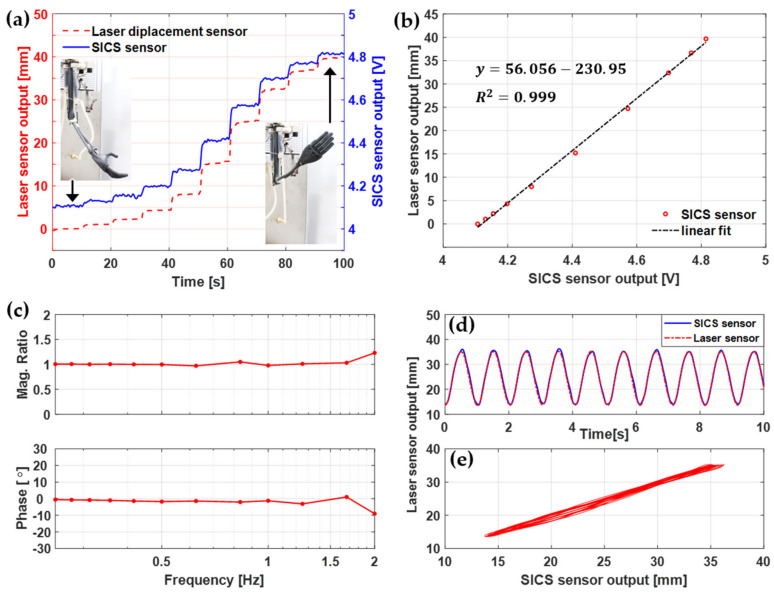
Comparisons of the laser displacement sensor output and SICS sensor output: (**a**) output comparison for the step motion of the robotic arm, (**b**) linear regression, (**c**) magnitude ratio and phase delay, (**d**) time-domain signals, and (**e**) Lissajous curve.

The outputs of the laser displacement sensor and SICS sensor were measured while supplying water of a constant temperature to the SI-SSBAs for 10 s and maintaining a specific strain of SI-SSBAs. This experiment was repeated while changing the water temperature from the maximum extension to the maximum flexion of the robotic arm, and the results are shown in Figure 8a. During the experiment, the measured SI-SSBA measured with a laser displacement sensor showed displacements of 0–40 mm, and the SICS sensor outputted voltages in the range of 4.1–4.8 V. The length of the SI-SSBA was 65 mm when the robotic arm was flexed and 105 mm when it was extended, generating a maximum extension of 61.5%. The SICS sensor outputs were stable in every section. Therefore, the designed SICS sensor can be used in the range of motion of the robotic arm. Furthermore, to acquire only stable output values excluding the section where the water temperature changes, the averages of the displacement of the laser displacement sensor and the output of the SICS sensor were calculated by extracting only the data between 2.5 and 7.5 s in 10 s when the temperature was maintained uniformly.

The relationship plotted between the displacement of the laser displacement sensor and the SICS sensor output is expressed as a first-order equation using the least squares method (Figure 8b). The coefficient of determination ($R^{2}$) was approximately 0.999, which is very close to that of the linear form. Each muscle experienced temperature changes of 54 °C from 36 to 90 °C. The output of the SICS sensor showing a linear relationship with the displacement of the laser displacement sensor indicated that the SICS sensor was not affected by the underwater operation environment and the temperature changes of 62 °C.

The measurement performance of the SICS sensor for the actuation speed of the SSBA was evaluated. The voltage output of the SICS sensor can be converted to the strain of the SI-SSBA using the first-order equation shown in Figure 8b. The displacement outputs of the laser displacement sensor and SICS sensor were measured for the strain of the SI-SSBA while increasing the flexion–extension speed of the robotic arm from 0.25 to 2 Hz. The magnitude ratio and phase difference were plotted by applying a fast Fourier transform to the two outputs. as shown in Figure 8c. The magnitude and phase of the two outputs were matched well until 1.67 Hz, but at 2 Hz, the SICS sensor output was delayed by 10° to the magnitude of 80%. This result confirmed that the SICS sensor has sufficient performance for application to the robotic arm because the target repetitive driving speed of the robotic arm driven by the SSBA is 1 Hz. Figure 8d,e shows the time data and Lissajous curve of the outputs of the laser displacement sensor and SICS sensor when the robotic arm was repeatedly driven at a speed of 1 Hz. The output of the SICS sensor is very similar to the output of the laser displacement sensor, although a slight hysteresis was observed.

## 6. Conclusions

This paper proposed a simple and soft inductive strain sensor fabricated using SMA coil springs, whose performance was verified through experiments. The SMA coil spring used as the sensor was fabricated using the same spring index and SMA wire as those of the SSBA. Thus, the SICS sensor has the advantage of generating strain while operating together with the SSBA, which is driven by the water temperature change, and measuring the strain of the SSBA without interrupting the motion of the SSBA. The linearity of the SICS sensor can be improved by placing the SICS sensor in an antagonistic structure. Our findings experimentally verified that by attaching the SI-SSBA fabricated by integrating the SSBA and SICS sensors to the robotic arm, the strain of the SSBA can be stably measured even at a driving speed of 1 Hz. Strain of the SSBA higher than 61.5% can be stably measured even in water with a temperature change of 54 °C from 36 to 90 °C. However, unlike arm motion, human fingers can move at speeds of 2 Hz or higher. Further research is needed to improve SICS sensor output at higher speeds.

This study was limited to the SICS sensor measuring only linear motion. However, it is also necessary to measure the effect of bending on the output of the SCIS sensor, which will be investigated in a future study.

The lightweight simple SICS sensor can be widely used to measure the strain of driven linear soft actuators, such as twisted polymer actuators and pneumatic actuators, and the strain of the SSBA. In addition, because the SICS sensor can be fabricated through an integration with a soft actuator, it can replace measurement sensors, such as encoders and LVDTs, in joints driven by soft actuators. In the future, we will conduct a study to improve the position control precision of robotic arms driven by the SSBA using the SICS sensor.

## Figures and Tables

**Table 1:** Specifications of the SMA coil spring for SICS.

Item	Value
SMA alloy	Nitinol (55% Ni)
Transition temperature, $A_{f}$	40 °C
SMA wire diameter	0.5 mm
Outer diameter of SMA coil spring	3.0 mm
SMA coil spring index	5
SMA coil spring mass	1.18 g
Number of coil spring turns	116
Vendor	Nexmetal, USA (https://www.nexmetal.com accessed on 22 February 2021)

## Data Availability

The data presented in this study are available on request from the corresponding author.

## Supplementary Materials

The following are available online at https://www.mdpi.com/1424-8220/21/7/2304/s1, Video S1: Robotic arm driven by SI-SSBA.
